# GISAID: Global initiative on sharing all influenza data – from vision to reality

**DOI:** 10.2807/1560-7917.ES.2017.22.13.30494

**Published:** 2017-03-30

**Authors:** Yuelong Shu, John McCauley

**Affiliations:** 1 WHO Collaborating Center for Reference and Research on Influenza, Chinese National Influenza Center, National Institute for Viral Disease Control and Prevention, Chinese Center for Disease Control and Prevention, Beijing, China; 2 WHO Collaborating Centre for Reference and Research on Influenza, Crick Worldwide Influenza Centre, the Francis Crick Institute, London, United Kingdom

**Keywords:** GISAID, Influenza, H7N9, H5N1, pandemic, data sharing

Ten years ago, a correspondence [1,2], signed by more than 70 championed ‘*A global initiative on sharing avian flu data*’ (GISAID) [3], leading to the GISAID Initiative in 2008. What started out as an expression of intent to foster international sharing of all influenza virus data and to publish results collaboratively has emerged as an indispensable mechanism for sharing influenza genetic sequence and metadata that embraces the interests and concerns of the wider influenza community, public health and animal health scientists, along with governments around the world. Today GISAID is recognised as an effective and trusted mechanism for rapid sharing of both published and ‘unpublished’ influenza data [4]. Its concept for incentivising data sharing established an alternative to data sharing via conventional public-domain archives.

In 2006, the reluctance of data sharing, in particular of avian H5N1 influenza viruses, created an emergency bringing into focus certain limitations and inequities, such that the World Health Organization (WHO)’s Global Influenza Surveillance Network (now the Global Influenza Surveillance and Response System (GISRS) [5]) was criticised on several fronts, including limited global access to H5N1 sequence data that were stored in a database hosted by the Los Alamos National Laboratories in the United States (US) [6,7]. This data repository, set up with financial support from the US Centers for Disease Control and Prevention (CDC) as a first attempt to share ‘sensitive’ data from affected countries, but was accessible only to those who were also providing H5N1 sequence data. This limited-access approach restricted wider sharing of data prior to publication, which was vital for broader understanding of the progress of the emergent public and animal health threat. The need for greater transparency in data sharing and for acknowledgement of those contributing samples from H5N1-infected patients and animals and related genetic sequence data was not satisfied by sharing data after formal publication via public-domain databases. Scientists charged with the day to day responsibilities of running WHO Collaborating Centres (CCs) for Influenza, National Influenza Centres and the World Organisation for Animal Health (OIE)/ Food and Agriculture Organization of the United Nations (FAO) [8] reference laboratories, were therefore eager to play a key role and provide scientific oversight in the creation and development of GISAID’s data sharing platform that soon became essential for our work.

A unique collaboration ensued, involving, in addition to members of WHO’s GISRS and OIE/FAO reference laboratories, the wider influenza research community along with officials in governmental institutions and non-governmental organisations. Facilitated by a well-connected broadcast executive with background in licensing of intellectual property, an agreement was drawn up on the sharing of genetic data to meet emergency situations, without infringing intellectual property rights - the GISAID Database Access Agreement (DAA). The DAA governs each individual’s access to and their use of data in GISAID’s EpiFlu database [9]. It was this alliance between scientists and non-scientists, with a diversity of knowledge and experience, involved in drawing up an acceptable simple, yet enforceable, agreement which gained the trust and respect of the scientific community and public health and animal health authorities.

The essential features of the DAA encourage sharing of data by securing the provider’s ownership of the data, requiring acknowledgement of those providing the samples and producing the data, while placing no restriction on the use of the data by registered users adhering to the DAA. It essentially defines a code of conduct between providers and users of data, cementing mutual respect for their respective complementary contributions, and upholding the collaborative ethos of WHO’s GISRS, initially established 65 years ago this year [5].

Launched in 2008, the EpiFlu database was of key importance in the response to the 2009 influenza A(H1N1) pandemic, allowing countries to readily follow the evolution of the new virus as it spread globally [10]. Acceptance of the GISAID sharing mechanism by providers and users of data, and the confidence of the influenza community, were further illustrated in 2013 by the unprecedented immediate release of the genetic sequences of Influenza A(H7N9) viruses from the first human cases, by Chinese scientists at the WHO Collaborating Centre for Influenza in Beijing [11,12]. Such events reaffirmed GISAID’s applicability to timely sharing of crucial influenza data. The subsequent use of the sequence data to generate, develop and test candidate vaccine viruses by synthetic biology within a few weeks also demonstrated how GISAID successfully bridged this important ‘technological’ gap [13,14]. The paper by Bao et al. from Jiangsu province of China published in this issue once again confirms the importance of the timely sharing of data on the evolution of the A(H7N9) viruses for global risk assessment. The authors analysed the recently isolated H7N9 viruses form the fifth wave in Jiangsu province, and the results showed no significant viral mutations in key functional loci even though the H7N9 viruses are under continuous dynamic reassortment and there is genetic heterogeneity. These findings should help to reduce concerns raised, even though the number of human infection with H7N9 virus increased sharply during the fifth wave in China.

GISAID provides the data-sharing platform particularly used by GISRS, through which sequence data considered by the WHO CCs in selecting viruses recommended for inclusion in seasonal and pre-pandemic vaccines are shared openly and on which research scientists, public and animal health officials and the pharmaceutical industry depend. Such openness of the most up-to-date data assists in an understanding of and enhances the credibility of the WHO recommendations for the composition of these seasonal and potential-pandemic vaccines.

Furthermore, in promoting the prompt sharing of data from potential pandemic zoonotic virus infections, as well as from seasonal influenza viruses, GISAID ensures a key tenet of the WHO Pandemic Influenza Preparedness (PIP) Framework [15], highlighting the critical role it plays in mounting an effective mitigating response. GISAID’s ability to facilitate efficient global collaborations, such as the Global Consortium for H5N8 and Related Influenza Viruses [16,17], is central to monitoring phylogeographic interrelationships among, for example, H5 subtype viruses in wild and domestic birds in relation to their incidence, cross-border spread and veterinary impact, and assessing risk to animal and human health [18].

Traditional public-domain archives such as GenBank, where sharing and use of data takes place anonymously, fulfil a need for an archive of largely published data; however, that conventional method of data exchange notably has not been successful in encouraging rapid sharing of important data in epidemic or (potential) pandemic situations, such as those caused by Middle East respiratory syndrome coronavirus (MERS-CoV) and Ebola viruses. While the GISAID EpiFlu database is hosted and its sustainability ensured through the commitment of the Federal Republic of Germany [19], the establishment of GISAID and development of the EpiFlu database was reliant to a large extent on philanthropy of one individual and voluntary contributions and generosity of many others, together with some initial financial provision by the US CDC and the German Max Planck Society.

That GISAID has become accepted as a pragmatic means of meeting the needs of the influenza community in part reflects the particular characteristics of influenza and the continual need for year-round monitoring of the viruses circulating worldwide, essential for the biannual vaccine recommendations and assessment of the risk posed by frequent zoonotic infections by animal influenza viruses [20]. In the meantime, calls for an equivalent mechanism to promote the timely sharing of data in other urgent epidemic settings go largely unfulfilled [21,22]. A recent publication considered whether the ‘paradigm shift’ in data sharing by GISAID could be applied more generally to assist in preparedness for and response to other emergent infectious threats, such as those posed by Ebola virus [21] and Zika virus [23]. Such a trusted system could complement and take full advantage of the latest advances in rapid sequencing of specimens in the laboratory and in the field, for outbreak investigation [24].

Given the crucial importance of genetic data in improving our understanding of the progress of an emergent, potentially devastating epidemic, the effectiveness of GISAID in influenza pandemic preparedness is self-evident and provides important lessons for future pandemic threats. While the genetic makeup and the necessary associated data of the different viruses are distinct requiring separate databases/compartments for unambiguous analysis, the modi operandi for sharing genetic data are generic and the GISAID mechanism could be applied to other emerging pathogens. Indeed, the wider implementation of such a data sharing mechanism should be key in concerted efforts to contain spread of disease in animals and threats to human health, in realising the concept of One Health.
